# EVA1A, a novel and promising prognostic biomarker in colorectal cancer

**DOI:** 10.3389/fonc.2024.1333702

**Published:** 2024-03-11

**Authors:** Hai-hua Fan, Hai-jun Zhang

**Affiliations:** ^1^ Department of Oncology, The Affiliated Tumor Hospital of Nantong University, Nantong, Jiangsu, China; ^2^ Department of Oncology, The Affiliated Zhongda Hospital of Southeast University, Medical School of Southeast University, Nanjing, Jiangsu, China

**Keywords:** colorectal cancer, Eva-1 homolog A, prognosis, biomarker, overall survival

## Abstract

**Purpose:**

The purpose of this study was to investigate the potential of EVA1A as a prognostic biomarker for Colorectal cancer (CRC).

**Methods:**

The study utilized public databases to analyze the difference in *Evala* mRNA expression between CRC tumor tissues and adjacent normal tissues. Additionallymunohistochemical staining was performed on 90 paired tissue samples to detect EVA1A expression. The relationship between EVA1A and clinicopathological features was examined, and a Kaplan-Meier survival analysis was conducted. Univariate and multivariate Cox analyses were employed to identify prognostic factors affecting the overall survival (OS) of CRC patients.

**Results:**

The analysis revealed a significant increase in *Evala* mRNA expression in CRC tumor cells compared to normal controls from public databases (P< 0.05). Immunohistochemical staining further confirmed a significant upregulation of EVA1A expression in CRC tissues (P< 0.05). High EVA1A expression was associated with age, pathological M stage, total tumor stage, and Carbohydrate antigen CA19-9 (CA19-9). Kaplan-Meier analysis demonstrated a significant association between high EVA1A expression and poor OS. Univariate and multivariate analysis identified EVA1A as an independent risk factor for CRC prognosis.

**Conclusion:**

The study suggests that EVA1A is increased in CRC tumor tissues and may serve as a potential biomarker for poor prognosis in CRC.

## Introduction

Colorectal cancer (CRC) is the most significant cause of mortality in men under the age of 50, ranking third in incidence and second in cancer-related deaths (1). Furthermore, since universal screening began in the mid-1990s, more patients have been diagnosed with the advanced stage. In China, approximately 20%-25% of patients are initially diagnosed with stage IV, which has poor clinical efficacy and a 5-year survival rate of fewer than 5% (2). Homologue of Eva-1 A [Evala, NCBI Gene ID: 84141, also known as Family with sequence similarity 176 (Fam176a), Transmembrane protein 166 (Tmem166)] is a new protein with cell and tissue type-specific expression that is well conserved in humans (3, 4). Recent studies have revealed that EVA1A expression is upregulated in liver and thyroid cancers, which might encourage tumor cell invasion and proliferation and result in a poor prognosis (5, 6). The link between EVA1A levels and CRC, on the other hand, is unknown.

As a result, the study examined the differential expression of EVA1A in CRC tissues and impartially assessed the clinical prognostic significance of EVA1A.

## Materials and methods

### Patients and tissue specimens

This study included 90 patients diagnosed with CRC at Zhongda Hospital, affiliated with the Medical School of Southeast University, from August 1, 2015, to August 1, 2020. These patients had detailed treatment histories and follow-up data. The cohort consisted of 48 males (53.33%) and 42 females (46.67%), with an average age of 67.68 ± 12.55 years, ranging from 28 to 93 years. The malignancies were located in the rectum (21 cases), right colon (37 cases), and left colon (32 cases). The pathological stages were determined based on the eighth edition of the American Joint Committee on Cancer (AJCC) Cancer Staging Manual (7), with 21 cases in stages I-II and 68 cases in stages III-IV.

After being discharged, all patients were closely monitored through phone calls and regularly asked to return to the hospital for checkups. The most recent follow up was completed on February 28, 2022, with a perfect 100% follow up rate. Overall Survival (OS) was defined as the time from the initial diagnosis of CRC to the unfortunate event of death, regardless of the cause. The study protocol was carefully designed to protect the private information of the enrolled patients under the stipulations of the Helsinki Declaration. In accordance with the ethical guidelines of the Ethics Committee of Zhongda Hospital, Southeast University, all patients were exempted from signing an informed consent form.

### Immunohistochemical staining

Ninety paired CRC tumors and normal tissues were procured from the pathology department of Zhongda Hospital to detect EVA1A expression. The majority of tumor tissues obtained from surgical specimens exhibited pathological features consistent with adenocarcinoma. Adjacent tissues were chosen as representative of normal tissue, and the reported findings were all intestinal mucosa. Pathological paraffin blocks were meticulously sectioned using an automatic paraffin microtome to produce pathological tissue sections of 4 μm thickness. These sections were stained with Hematoxylin and Eosin (HE) using an automatic staining machine. The HE-stained sections underwent a series of processes, including baking, deparaffinization, antigen repair, antibody incubation, counterstaining with hematoxylin, sealing, and microscopic examination.

**Figure 1:**
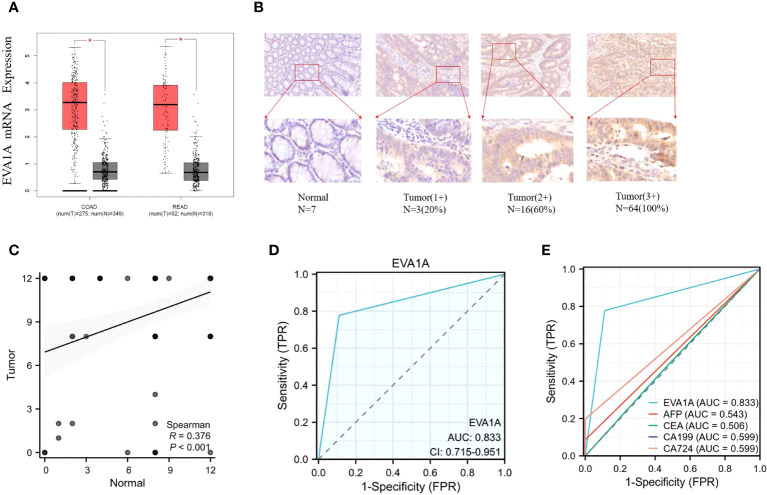
Expression of EVA1A in CRC tumor and normal tissues. **(A)** Differential expression of Eva1a mRNA between CRC tumor and normal tissues. **(B)** Immunohistochemical results of typical normal tissues and tumor tissues with different staining intensities. **(C)** Differential expression of EVA1A between CRC tumor and normal tissues. **(D)** EVA1A represented a smart diagnostic value. **(E)** ROC for the diagnostic efficiency of EVA1A, serum AFP, serum CEA, serum CA19–9, and serum CA72-4. *p<0.05.

The positive reaction of immunohistochemical staining was identified as yellow-tan particles (**Figure 1B**). Initially, a representative area was observed under a low-power microscope (100×), followed by counting 500-1000 tumor cells under a high-power microscope (400×). We employed the semi-definite optimal integration method to assess the outcome. The results were independently evaluated by two pathologists based on the staining intensity and the percentage of positive cells. The staining intensities were categorized as 0 (negative), 1 (positive 1+), 2 (positive 2+), and 3 (positive 3+), respectively. The percentages of cells were classified as 0 (negative), 1 (1–25%), 2 (26–50%), 3 (51–75%), and 4 (76–100%), respectively (8, 9). The scoring system encompassed a range from 0 to 12, yielding a mean value of 10. We designated this value as the threshold for staining, with ≤10 indicating negativity (Low expression group) and >10 indicating positivity (High expression group).

### Statistical analysis

Statistical analyses and mapping were performed using SPSS software (version 26.0, IBM Corporation, Armonk, NY, USA), GraphPad Prism (Version 8.4.3, GraphPad Software, La Jolla, CA, USA), and R (version 3.4.1, http://www.r-project.org/) in the present study. The association between EVA1A expression and clinicopathological characteristics was investigated using the χ2 test and continuity correction, while the Wilcoxon test was employed to assess significant differences between CRC tumors and normal tissues. The receiver operating characteristic (ROC) curves for CRC were utilized to examine the diagnostic efficacy of EVA1A expression. At an ideal threshold, the sensitivity and specificity were assessed. Based on the cutoff, the expression of EVA1A was categorized as high or low. The Kaplan-Meier curve was used to examine survival data, and the Log-rank test was used to evaluate group differences. For the survival analysis, both univariable and multivariable analyses were applied. Multivariate analysis could be performed with variables whose univariate analysis(P< 0.1) findings indicated significant associations. P< 0.05 was considered to be statistically significant.

## Results

### EVA1A expression in CRC tissues

The expression of EVA1A was analyzed using the GEPIA database(GEPIA (cancer-pku.cn)), which is publicly accessible. The analysis included data from 367 patients with tumor and 667 normal tissue samples. The data was sourced from The Cancer Genome Atlas (TCGA) and the Genotype-Tissue Expression (GTEx) databases. The analysis revealed a significant difference in the expression of *Eva1a* mRNA between the two groups. Additionally, there was a higher expression of *Eva1a* mRNA in CRC tumor tissues than in normal tissues (P< 0.05, **Figure 1A**).

Subsequently, the protein expression of EVA1A was examined in tumor tissues (n=90) and normal tissues (n=90) via immunohistochemistry staining conducted at our institution. The results, shown in **Figure 1B**, indicate that EVA1A is mainly located in the cytoplasm. Additionally, EVA1A was found to be overexpressed in CRC (P< 0.001, **Figure 1C**). **Table 1** enumerates the number of patients categorized by different scores based on immunohistochemistry staining. These findings collectively suggest that EVA1A is upregulated in CRC.

**Table 1:** The number of patients in different scores based on immunohistochemistry staining scores

Scores	Tumor	Normal
0	7	5
1	1	2
2	3	7
3	0	1
4	1	4
6	0	2
8	14	24
9	0	1
12	64	44

### Diagnostic value of EVA1A expression in CRC

We conducted a study to assess the diagnostic potential of EVA1A expression in CRC. We performed a Receiver Operating Characteristic (ROC) analysis on the scores of CRC tumor tissues and normal tissues, as shown in **Figures 1D, E**. When the cut-off value is 0.5, the Area Under the Curve (AUC) value of EVA1A is observed to be 0.833, its sensitivity is 0.778 and specificity is 0.889, which is higher than the values associated with alpha-fetoprotein (AFP), carcinoembryonic antigen (CEA), carbohydrate antigen 19-9 (CA19-9), and carbohydrate antigen 72-4 (CA72-4). The ROC-related information and data for each predictor at their optimal cut-off values are presented in **Table 2**.

**Table 2:** ROC-related information and data.

Variable	AUC	C.I.	Cut-off value	Sensitivity	Specificity	Youden-index
EVA1A	0.833	0.715-0.951	0.5	0.778	0.789	0.667
AFP	0.543	0.512-0.574	0.5	0.0868	1	0.086
CEA	0.506	0.326-0.687	0.5	0.457	0.556	0.012
CA199	0.599	0.555-0.642	0.5	0.198	1	0.198
CA724	0.599	0.555-0.642	0.5	0.198	1	0.198

### EVA1A expression in CRC with different clinicopathological variables

To investigate the relationship between EVA1A and CRC, we stratified CRC patients into two distinct cohorts: those exhibiting low EVA1A expression (score 0-10, n=26) and those demonstrating high EVA1A expression (score 10-12, n=64). **Table 3** provides a comprehensive summary of the correlation between EVA1A expression and various clinicopathological factors relevant to CRC. Notably, the patient’s chronological age, the pathological M stage, the aggregate tumor stage, and the CA199 marker were significantly intertwined with EVA1A expression (P< 0.05).

Table 3Relationships between the expression level of EVA1A and the clinicopathological characteristics of CRC patients (The reference range of characteristics was based on the Laboratory Department of Zhongda Hospital, Southeast University).

**Table 3-1:** Univariate analyses of associations between EVA1A and clinicopathological characteristics.

Characteristics		Low-group (N=36)	High-group (N=64)	χ2*/Fisher* test	*P* value
**Age(years)**	<=65	14	19	4.647	0.031
	>65	12	45		
**Sex**	Male	11	37	1.786	0.181
	Female	15	27		
**Hypertension**	No	16	30	1.591	0.267
	Yes	10	34		
**Diabetes**	No	22	58	0.205	0.651
	Yes	4	6		
**Smoking**	No	20	60	3.734	0.053
	Yes	6	4		
**Drinking alcohol**	No	21	59	1.421	0.233
	Yes	5	5		
**Tumor location**	Right colon	9	28	0.664	0.717
	Left colon	10	22		
	Rectum	7	14		
**Pathological type**	Adenocarcinoma	22	54	0	1.000
	Non- adenocarcinoma	4	10		
**Differentiation**	Poorly differentiated	5	10	0.560	0.756
	Moderately differentiated	21	53		
	Well differentiated	0	1		
**T stage**	T1	1	4	1.107	0.775
	T2	2	2		
	T3	22	56		
	T4	1	2		
**N stage**	N0	10	14	4.24	0.120
	N1	12	28		
	N2	4	22		
**M stage**	M0	24	48	3.462	0.063
	M1	2	16		
**Grade**	I-II	10	11	4.687	0.031
	III-IV	16	53		
**AFP**	<=7ng/mL	26	57	1.747	0.186
	>7ng/mL	0	7		
**CEA**	<=5ng/mL	16	34	0.530	0.467
	>5ng/mL	10	30		
**CA199**	<=39U/mL	24	47	3.953	0.047
	>39U/mL	2	17		
**CA724**	<=6.9U/mL	24	48	3.462	0.063
	>6.9U/mL	2	16

**Table 3-2:** Multivariate analyses of associations between EVA1A and clinicopathological characteristics.

Characteristics	B	S.E	Wals	df	*P* value	Exp (B)	95%C.I.
**Age**	1.1 33	0.502	5.082	1	0.024	0.322	0.120-0.863
**Grade**	1.2 34	0.548	5.062	1	0.024	0.291	0.099-0.853

### Relationship between EVA1A expression and OS in CRC

**Figure 2:**
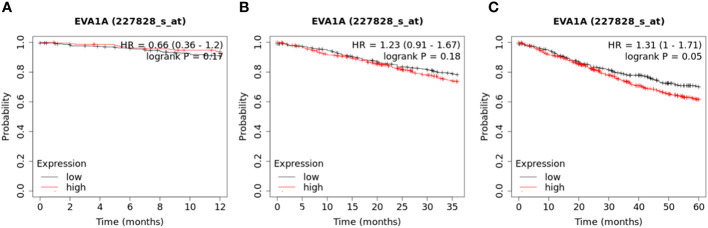
Kaplan–Meier curves of overall survival in 809 CRC patients with high and low Eva1a mRNA expression (https://kmplot.com/analysis/index.php?p=service&cancer=colon). These patients were divided into two groups based on the median value of EVA1A expression. **(A)** 1-year survival rate (Log-rank Mantel-Cox) : P=0.17; **(B)** 3-year survival rate (Log-rank Mantel-Cox) : P=0.18; **(C)** 5-year survival rate (Log-rank Mantel-Cox) : P=0.05.

Survival data was obtained from the dataset for a cohort of 809 CRC patients. (https://kmplot.com/analysis/index.php?p=service&cancer=colon). These patients were divided into two groups based on the median value of EVA1A expression to explore their 1 -, 3 -, and 5-year survival rates. A significant correlation was found between high EVA1A expression and reduced overall survival in CRC patients (**Figure 2**).

**Figure 3:**
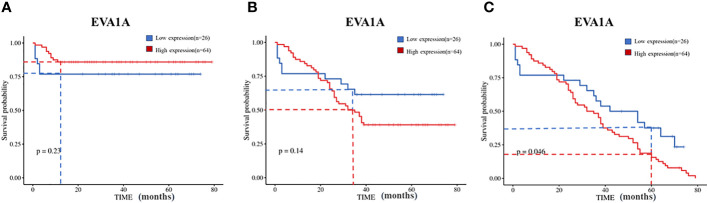
Kaplan–Meier curves of overall survival in 90 CRC patients with high and low Eva1a mRNA expression. **(A)** 1-year survival rate (Log-rank Mantel-Cox) : χ2=1.470, P=0.23; **(B)** 3-year survival rate (Log-rank Mantel-Cox) : χ2=2.185, P=0.14; **(C)** 5-year survival rate (Log-rank Mantel-Cox) : χ2=3.965, P=0.046.

Additionally, our institution used the Kaplan-Meier method to analyze the relationship between EVA1A protein expression and overall survival rates. The 1, 3, and 5-year overall survival rates were 83.3% (75/15), 45.6% (41/49), and 10% (9/81) respectively. It was discerned that patients with low EVA1A expression had significantly protracted survival times compared to those with high EVA1A expression (**Figure 3**).

### EVA1A overexpression as an independent risk factor in CRC

**Figure 4:**
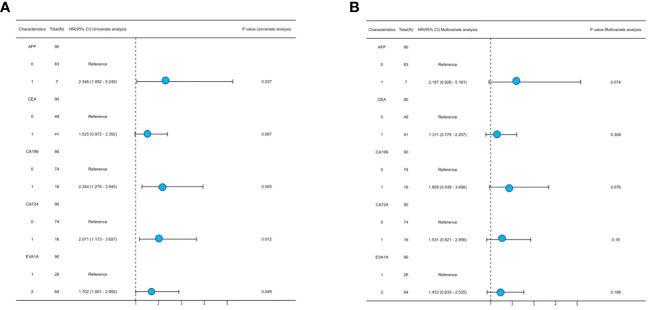
Forest plot of univariate **(A)** and multivariate **(B)** cox regression.

Both univariate and multivariate Cox regression analyses were performed to identify prognostic factors that affect the OS in CRC patients. The results of the univariate Cox regression analysis showed that the expression of EVA1A (P= 0.049) was found to be an independent risk factor, indicating a poor prognosis for CRC (**Table 4**). As depicted in **Figure 4**, the forest plot provides a visual representation of the specific hazard ratios associated with these risk factors.

**Table 4:** Univariate and multivariate analysis of clinicopathological characteristics affecting prognosis of patients with CRC.

Characteristics	Total(N)	Univariate analysis			Multivariate analysis	
		Hazard ratio (95% CI)	P value		Hazard ratio (95% CI)	P value
**AFP**	90					
Low	83	Reference			Reference	
High	7	2.348 (1.052 - 5.240)	0.037		2.187 (0.926 - 5.161)	0.074
**CEA**	90					
Low	49	Reference			Reference	
High	41	1.525 (0.972 - 2.392)	0.067		1.311 (0.779 - 2.207)	0.308
**CA199**	90					
Low	74	Reference			Reference	
High	16	2.244 (1.276 - 3.945)	0.005		1.859 (0.938 - 3.686)	0.076
**CA724**	90					
Low	74	Reference			Reference	
High	16	2.071 (1.173 - 3.657)	0.012		1.531 (0.821 - 2.856)	0.180
**EVA1A**	90					
Low	26	Reference			Reference	
High	64	1.702 (1.001 - 2.892)	0.049		1.453 (0.833 - 2.535)	0.188

## Discussion

In China, colorectal cancer presents a significant health burden, with an alarming 387,600 new diagnoses and 187,100 fatalities annually (10). The pathogenesis and progression of CRC is a complex, multi-stage process that incrementally transitions from benign mucosa and adenoma to malignant carcinoma. Serum tumor markers serve as indispensable tools for the early identification of malignancies, aiding in tumor staging and prognostic evaluation. In a clinical setting, the frequently employed serum tumor markers for CRC encompass AFP, CEA, CA19-9, and CA72-4, among others. However, clinical observations have underscored certain limitations in their diagnostic sensitivity and specificity (11). Consequently, it is crucial to explore the causes and mechanisms of CRC to discover new tumor markers. In this investigation, we have elucidated that EVA1A is markedly overexpressed in CRC, making it a reliable prognostic indicator for CRC patients. In instances where the AUC exceeds 0.5, the closer the AUC approaches 1, the more robust the diagnostic efficacy of the variable in predicting outcomes. Accuracy is diminished when the AUC ranged from 0.5 to 0.7, improved when the AUC ranges from 0.7 to 0.9, and further enhances when the AUC surpasses 0.9. Notably, when AUC equals 0.5, the variable exhibits no discernible effect and lacks diagnostic value. Our findings show that EVA1A demonstrates an AUC of 0.833, surpassing that of AFP, CEA, CA199, and CA724, thereby exhibiting commendable accuracy and clinical utility.

EVA1A, an enigmatic lysosomal and endoplasmic reticulum-associated protein, pervades most human tissues and organs, bearing no discernible homology to any recognized gene or protein (12). An accumulating body of evidence implicates its association with tumorigenesis and cancer progression. Contemporary research has unveiled that the prevalence of EVA1A protein expression in T3/T4 stage esophageal squamous cell carcinoma stands at a substantial 77.8% (21/27), markedly surpassing that in the T1/T2 stage (41.7%, 5/12). This disparity is statistically significant (P< 0.05), thereby suggesting a positive correlation between EVA1A expression levels and the extent of tumor invasion (13). The results of this study support the established hypothesis that the high expression of EVA1A in CRC tissues is significantly correlated with the M stage and overall stage of the tumor, with a statistically significant difference (P< 0.05). This suggests that EVA1A may play a role in the initiation and progression of CRC. EVA1A is a potentially novel protein involved in regulating autophagy and apoptosis. Previous research has shown that EVA1A can enhance resistance to the drug oxaliplatin in liver cancer by inducing autophagy (14).

Further analysis has revealed that autophagy may increase drug resistance and enhance drug sensitivity in cancer (15, 16). The apparent contradiction in these findings may be due to the specific type of cancer or the specific drug used. Therefore, more research is needed to understand the role of autophagy in EVA1A-related drug resistance in tumors. Furthermore, a body of research suggests that EVA1A could potentially stimulate the proliferation and metastasis of papillary thyroid cancer (PTC) by impeding apoptosis (6), bolstering the Hippo signaling pathway, and instigating Epithelial-mesenchymal transition (EMT). This could represent a pivotal mechanism through which EVA1A influences malignant progression. Moreover, a significant correlation was observed between EVA1A and the prognosis of PTC with lymph node metastasis (P=0.038< 0.05), establishing it as an independent prognostic factor. This offers a promising avenue for immunotherapy to thwart the advancement of thyroid (5).

Within the intricate landscape of oncological advancement, autophagy is perceived as a paradoxical phenomenon akin to a ‘double-edged sword.’ Autophagy possesses the capacity to decelerate the demise of CRC tumor cells, thereby engendering a formidable resistance to therapeutic interventions (17). In their study, Wu and colleagues found that increased autophagy levels may contribute to the survival and invasiveness of CRC (18). They also observed that the amplification of EVA1A expression stimulates the proliferation of the autophagic membrane and the maturation of autophagosomes by enhancing the binding between Autophagy Related 5 (ATG7) and the ATG5/ATG12 complex. This process potentially promotes metastasis within the cancerous environment (12). However, the exact mechanism by which autophagy facilitates EVA1A in promoting the malignant transformation of CRC remains unknown and requires further investigation in future studies.

In this study, we investigated the relationship between the expression of EVA1A and the invasive characteristics and prognosis in CRC progress. Our findings confirm that EVA1A is a reliable prognostic biomarker and a potential therapeutic target for CRC. Previous research by several scholars has analyzed 16 survival-associated autophagy genes (ARGs), including EVA1A, FOXO1, and HIF1, to assess the risk and adverse prognosis of multiple myeloma (MM). They discovered that MM patients with decreased EVA1A expression had significantly reduced survival duration (P=0.006< 0.05) (19). Similarly, in a longitudinal study of CRC patients, elevated EVA1A expression was associated with a poor prognosis. In addition, in TCGA database, a significant correlation was found between high EVA1A expression and reduced overall survival in 597 CRC patients (P=0.0041< 0.05) (Expression of EVA1A in colorectal cancer - The Human Protein Atlas). These findings suggest that EVA1A presence in CRC is a risk factor, consistent with existing literature. Additionally, an excess of EVA1A seems to promote tumor progression. However, further research is needed to explore the precise regulatory mechanisms and the impact of EVA1A on CRC cell proliferation, invasion, and metastasis *in vitro*.

Furthermore, research has revealed that EVA1A partially colocalizes with lipid droplets (20), suggesting a potential link to lipid metabolism in CRC. This discovery provides a starting point for exploring the development of CRC. Ming-Tao and his team demonstrated that EVA1A plays a role in the formation and advancement of pancreatic tumors, highlighting its significant presence in the α cells of normal pancreatic tissue. This suggests a potential regulatory influence on α cell function (21). However, the specific impact and underlying mechanism require further investigation.

This study, although informative, has limitations. It was mainly conducted in a single institution, so generalizing its findings to a broader range of hospitals requires additional validation, considering the potential impact of selection bias. Additionally, the small size of the study group may result in false negative results. In the future, it is crucial to increase the sample size and conduct prospective studies to address the inherent heterogeneity of neoplasms.

## Conclusion

In conclusion, we found increased EVA1A expression in CRC tissues, closely associated with patient age, tumor metastasis and stage, and the CA199 marker. Our study highlights EVA1A’s role as an independent prognostic risk factor for CRC patients. The importance of EVA1A as a potential biomarker, offering clinicians a valuable tool to improve patient monitoring and increase survival rates.

## Data availability statement

The original contributions presented in the study are included in the article/supplementary material. Further inquiries can be directed to the corresponding author.

## Ethics statement

The studies involving humans were approved by The Ethics Committee of Zhongda Hospital, Southeast University. The studies were conducted in accordance with the local legislation and institutional requirements. The human samples used in this study were acquired from primarily isolated as part of your previous study for which ethical approval was obtained. Written informed consent for participation was not required from the participants or the participants’ legal guardians/next of kin in accordance with the national legislation and institutional requirements. Written informed consent was obtained from the individual(s) for the publication of any potentially identifiable images or data included in this article.

## Author contributions

H-HF: Writing – original draft, Investigation, Methodology, Data curation, Formal analysis, Software, Visualization. H-JZ: Writing – review & editing, Resources, Supervision, Conceptualization, Validation.
